# Ear, Nose, and Throat Foreign Bodies in Children: A Retrospective Study

**DOI:** 10.3390/children9010063

**Published:** 2022-01-04

**Authors:** Bin Kwon, Yeso Choi, Sung-Kyun Kim, Seok-Jin Hong, Yong-Bok Kim, Seok-Min Hong

**Affiliations:** Department of Otorhinolaryngology—Head and Neck Surgery, Dongtan Sacred Heart Hospital, Hallym University College of Medicine, Hwaseong 18450, Korea; binkwon@hallym.or.kr (B.K.); 201080@hallym.or.kr (Y.C.); newearera@hallym.or.kr (S.-K.K.); enthsj@hallym.or.kr (S.-J.H.); yongbok@hallym.or.kr (Y.-B.K.)

**Keywords:** foreign body, children

## Abstract

Background: This study analyzed the presentation, characteristics, and management of foreign bodies in different age groups of pediatric patients with ear, nose, and throat foreign bodies. Methods: A retrospective study was performed using data from October 2012 to September 2020. A total of 1285 patients with ear, nose, and throat foreign bodies who were less than 12 years of age and who presented to the emergency room were included in this study. Their biographical data, clinical presentations, foreign body types and locations, and management outcomes were obtained from medical records and analyzed as three age groups (infancy: <2 years old; early childhood: 2–5 years old; and late childhood: 6–12 years old). Results: The early childhood group had the highest number of patients (*n* = 672; 52.2%). Throat was the most common location (59.2%), and bone was the most common type of foreign body. Among the children who visited our hospital, foreign bodies were actually found in only 657 patients (51.1%) and removed by an otolaryngologist in 625 (95.1%) cases. Conclusion: Our study could provide guidance for the diagnosis and management of pediatric patients who present to emergency departments with foreign bodies.

## 1. Introduction

Foreign bodies in the ear, nose, and throat are a common problem encountered by not only otorhinolaryngology-head and neck surgeons but pediatrics or family medicine doctor in clinics and emergency departments [1,2,3]. Children often present to emergency rooms when their parents find or suspect foreign bodies in their ears, nose or throat [4,5]. Children undergoing a stage of exploratory development have a tendency to place objects in their ears, nose, and mouth. These children show either atypical symptoms, such as crying, non-specific discomfort, refusal to eat, and presence of blood-stained saliva, or no symptoms at all [3,6].

Unlike adults, children are often uncooperative, which makes the diagnosis and treatment of foreign bodies difficult for clinicians [3]. Prompt recognition and management are required to minimize complications and mortality (although very rare), even if the foreign body is not found [7,8]. Previous studies conducted on a small scale have analyzed the locations of ear, nose, and throat foreign bodies [3,4,5,9,10]. However, it is important to comprehensively analyze the general characteristics of foreign bodies in children.

In this study, we reviewed a large number of pediatric patients who presented to the otorhinolaryngology department through the emergency room, with foreign bodies in the ear, nose, and throat, including the pharynx and larynx. The patients were divided into three groups (infancy: <2 years old; early childhood: 2–5 years old; and late childhood: 6–12 years old) to investigate the characteristics based on their ages.

## 2. Materials and Methods

### 2.1. Subjects and Clinical Variables

This study was approved by our Institutional Review Board (2020-11-006). All procedures were performed in accordance with the Declaration of Helsinki.

We retrospectively reviewed the medical records of pediatric patients (<12 years) with foreign bodies between 31 October 2012, and 30 September 2020, who presented to the emergency room of Hallym University Dongtan Sacred Heart Hospital for a foreign body in the ear, nose, and throat. We selected 1285 patients who met the inclusion criteria.

### 2.2. Classification and Foreign Body Analysis

After the child was seated in an otolaryngology chair, the head was instructed not to move, and the ears, nose, and throat were observed using the naked eye or endoscope. In the case of a foreign body in the nose, cotton pledget including 1:100,000 epinephrine was placed in the nasal cavity for 5 min to improve visibility, and then removed and observed. If a foreign body was observed, it was removed using suction, bayonet forceps, alligator forceps, tonsil kelly, pick, and steel ear tips. If foreign body removal from the throat was unsuccessful, or if a foreign body was suspected but unverified, the patient was referred to a physician for esophagogastroscopy. Foreign bodies were removed under general anesthesia in an operating room if there was a risk of complications, anatomical problems, or poor compliance.

The patients were divided into three age groups (infancy: <2 years; early childhood: 2–5 years; and late childhood: 6–12 years). The number of patients with foreign bodies in each age group was determined. Foreign body locations, types, presence or absence, removal methods, and complications were reviewed in each age group.

The foreign bodies were classified based on their location (ear, nose, or throat foreign bodies) and type (food, toy or play instruments, bones, insects, and other). The presence or absence of foreign bodies, removal methods, and complications were determined using medical records.

## 3. Results

### 3.1. Demographic Data

There were 591 females (46.0%) and 694 males (54.0%) among the 1285 patients. The early childhood group had the highest number of patients (*n* = 672; 52.2%) followed by the late childhood (*n* = 514; 40.0%) and infancy (*n* = 99; 7.8%) groups (Table 1).

### 3.2. Clinical Data

The most common foreign body location was the throat (*n* = 761; 59.2%), followed by the nose (*n* = 429; 33.4%) and ears (*n* = 95; 7.4%). In the infancy and late childhood groups, throat foreign bodies were the most common (61.6% and 74.9%, respectively). In the early childhood group, throat and nose foreign bodies had similar frequencies (48.3% and 46.8%, respectively).

Bones (*n* = 609; 47.4%) were the most common type of foreign bodies, followed by toys, such as buttons, clay, and pencils (*n* = 319; 24.8%), and foods, such as peels, kernels, seeds, and medicines (*n* = 185; 14.4%). In the infancy group, foods, bones, and toys (21.2%, 23.2%, and 23.2%, respectively) had similar frequencies. However, the frequency of bone-type foreign bodies increased with increasing age (38.0% and 64.2% in the early and late childhood groups, respectively) (Table 2).

Toys accounted for more than half of the foreign bodies in the ears and nose. In the nose, foods were the second-most common foreign body type (23.5%). In the throat, bones were the most common foreign bodies, followed by foods and toys (79.8%, 9.1%, and 7.4%, respectively) (Table 3).

Foreign bodies were actually found by the doctor in 657 (51.1%) of the 1285 patients. The infancy group had the lowest rate of foreign body retrieval, followed by the late and early childhood groups (40.4%, 49.6%, and 53.8%, respectively).

An analysis of the presence or absence of foreign bodies on the basis of location showed that foreign bodies were found in the ear in 83 patients (87.4%) and in the throat in 279 patients (36.7%) (Table 4).

Foreign bodies were removed by an ear, nose, and throat doctor in the emergency room in 625 (95.1%) of 657 patients. Foreign bodies were removed using esophagogastroscopy in 4 patients (0.6%), and under general anesthesia in an operating room in 28 patients (4.3%). 

Foreign bodies in the ear required removal under general anesthesia more frequently (*n* = 15/83; 18.1%) compared to those in the throat (*n* = 7/280; 2.5%) and nose (*n* = 6/294; 2.0%) (Table 5).

Complications occurred in only 4 (0.3%) patients; 1 (1%) in infancy (epistaxis), and 3 (0.5%) in the late childhood (tympanic membrane perforation, external auditory canal injury, and laryngeal mucosal injury) group (Table 6).

## 4. Discussion

Several studies have demonstrated a higher frequency in children under 5 years of age, in agreement with our findings [3,9,10]. A previous study reported that the ear was the most common location, but we found that the throat was the most common location across all age groups [2].

We divided the children into three age groups and found that foreign bodies were most common in the early childhood group. Other study showed that age between 1–2 years old were most frequent age group who visited hospital for foreign bodies [11]. Children learn to walk around the age of two years, which allows them to evade parental observation and introduce foreign objects in their ears, nose, and throat.

Bones were the most frequently found foreign bodies in children, being more common in the early and late childhood groups. Patients with throat foreign bodies presented to the hospital with symptoms of a foreign body sensation and throat pain. Most of the foreign bodies were found in the oropharynx or hypopharynx. Previous studies have reported bones, peanuts, and other types of nuts as the most common foreign bodies [2,3,8,12]. Fish and other seafood, such as shellfish and crab, are commonly consumed in Korea. Therefore, we classified bones separately from other foods. The types and frequencies of foreign bodies may vary with types of foods, depending upon the race, culture, and environment of the study subjects. In other studies conducted in Asia, fish bones were most frequent foreign body [13,14]. Patients with foreign bodies in the nose presented with symptoms of nasal congestion, nasal discomfort, and a runny nose. The most frequently found foreign bodies in the nose were toys, followed by foods. Interestingly, the number of foreign bodies in the nose was highest in the early childhood group, although there is no significant difference compared to the number of foreign bodies in the throat. Children develop a tendency to place objects into their mouth by the age of six months. This tendency disappears by early childhood, and there seems to be a tendency for children to put toys and foods in their noses. Patients with a foreign body in the ears presented with symptoms of ear pain and stuffiness. Contrary to previous studies, which found insects and cotton swabs as the most common foreign bodies, we found toys (e.g., buttons, clay, pencils, crayons, stickers, marbles, paper, and magnets) to be the most common foreign bodies, while insects were the fourth most common foreign bodies [2,9].

We found that only 657 (51.1%) patients actually had a foreign body, and no foreign bodies were found in the remaining 629 patients. It is possible that the foreign body causing the discomfort may have been removed spontaneously; foreign bodies in the throat may easily pass through the esophagus. In our study, the detection rates of foreign bodies were the lowest in the throat.

Most of the foreign bodies could be removed in outpatient clinics [6,9,10]. Foreign bodies were detected visually or endoscopically. If a foreign body could not be detected despite symptoms, or if the removal was difficult, an internal medicine doctor was asked to perform esophagogastroscopy. Foreign bodies were removed in the operating room under general anesthesia (*n* = 28/657; 4.3%) if the child was uncooperative, if there was a risk of complications, or if the removal was unsuccessful. In particular, a foreign body in the ears often required general anesthesia (*n* = 15/95; 15.8%). On the other hand, general anesthesia was rarely required for nose (*n* = 9/429; 1.4%) and throat (*n* = 7/761; 0.9%) foreign bodies. Children have narrower external auditory canals and greater amounts of earwax compared to adults. They are also often uncooperative, which makes removal difficult and increases the risk of injuring surrounding structures. This is the reason that general anesthesia is often required. Moreover, the risk of eardrum damage increases if the foreign body is in close proximity. Foreign body removal from the nose and throat is relatively easier and foreign bodies may be removed spontaneously by physiological mechanisms, such as coughing, sneezing, nausea, or a runny nose [1]. Therefore, general anesthesia is not commonly required. 

Additional esophagogastroscopy was performed by internal medicine in four cases for the removal of foreign bodies in the throat. It is difficult for children to express their symptoms clearly, and they may be ignored by parents or doctors. If a foreign body is strongly suspected but not found, esophagogastroscopy may be considered after gaining informed consent from the parents or guardians, even if the child is unwilling.

Complications occurred in 4 out of 1285 patients, which is much lower than in other studies [1,2]. Minor problems, such as wounds or minor bleeding during the removal process, were not considered as complications in this study. However, unlike other studies, there were no severe complications, such as peritonsillar abscesses or infections [2,6]. In the infancy group, there was one case of epistaxis in the nose foreign body, which required treatment (digital pressure or a vasoconstrictor-soaked pledget). Other foreign objects included beans (48 patients) and batteries (3 patients). Beans can get swollen in the nasal cavity and cause pressure on the adjacent nasal mucosa, and may result in septal necrosis, infection, or perforation. Batteries can cause chemical reactions that may result in septal perforation. In our study, there were no septal perforations, necrosis, or infections resulting from beans and batteries. It is thought that this is because we removed foreign bodies in a relatively short time from patients who visited the emergency room. In the ear foreign bodies, eardrum perforation and external ear canal injury occurred in one patient each, both in the late childhood group. The frequency of these complications was also lower compared to that in previous studies [9]. In the study, the frequency of insects was higher in the ears compared to the other sites, and complications was thought to be more likely during insect removal, including a dead carcass or eggs. In our study, there were ten patients with insects in the ear, but only one had complications (eardrum perforation). Since children in late childhood are relatively larger and stronger, uncooperative children were immobilized by the medical staff, increasing the risk of injuring the surrounding tissues during foreign body removal.

## 5. Conclusions

Although this was a retrospective study, foreign bodies were investigated in a large number of pediatric patients. Foreign bodies were most common in the early childhood group, and they showed various clinical characteristics on the basis of location and the patient’s age. Our data may provide clinical information to doctors managing pediatric foreign body patients.

## Figures and Tables

**Table 1:** Demographic data.

Characteristic	n	Percentage (%)
Sex		
Males	694	54.0
Females	591	46.0
Age groups		
<2 years	99	7.8
2–5 years	672	52.2
>5 years	514	40.0
Total	1285	100

**Table 2:** Location and type of foreign body in the age group.

Age	<2 Years (%)	2–5 Years (%)	>5 Years (%)	Total (%)
Location				
Ear	2 (2.0)	32 (4.8)	61 (11.8)	95 (7.4)
Nose	36 (36.4)	325 (48.4)	68 (13.2)	429 (33.4)
Throat	61 (61.6)	315 (46.8)	385 (75.0)	761 (59.2)
Type				
Foods	21 (21.2)	105 (15.6)	59 (11.5)	185 (14.4)
Bones	23 (23.2)	256 (38.1)	330 (64.2)	609 (47.4)
Toys	23 (23.2)	209 (31.1)	87 (16.9)	319 (24.8)
Insects	0 (0)	4 (0.6)	7 (1.4)	11 (0.9)
Other	32 (32.3)	98 (14.6)	31 (6.0)	161 (12.5)
Total	99	672	514	1285

**Table 3:** Types of foreign bodies on the basis of location.

Location	Throat (%)	Nose (%)	Ear (%)	Total (%)
Type				
Foods	70 (9.2)	101 (23.5)	14 (14.7)	185 (14.4)
Bones	608 (79.9)	0 (0.0)	1 (1.1)	609 (47.4)
Toys	26 (3.4)	239 (55.7)	54 (56.8)	319 (24.8)
Insects	0 (0.0)	0 (0.0)	11 (11.6)	11 (0.9)
Other	57 (7.5)	89 (20.7)	15 (15.8)	161 (12.5)
Total	761	429	95	1285

**Table 4:** Presence of foreign bodies in the age groups and location.

Presence	Yes (%)	No (%)	Total
Age			
<2 years	40 (40.4)	59 (59.6)	99
2–5 years	362 (53.9)	310 (46.1)	672
>5 years	255 (49.6)	259 (50.4)	514
Location			
Throat	279 (36.7)	482 (63.3)	761
Nose	295 (68.8)	134 (31.2)	429
Ear	83 (87.4)	12 (12.6)	95
Total	657 (51.1)	629 (48.9)	1285

**Table 5:** Methods of foreign body removal in the age groups and location.

Presence	ER (%)	EGD (%)	OR (%)	Total
Age				
<2 years	35 (89.7)	3 (7.7)	1 (2.6)	39
2–5 years	335 (94.1)	1 (0.3)	20 (5.6)	356
>5 years	255 (97.3)	0 (0.0)	7 (2.7)	262
Location				
Throat	269 (96.1)	4 (1.4)	7 (2.5)	280
Nose	4 (1.4)	0 (0)	6 (2.0)	294
Ear	7 (2.5)	0 (0)	15 (18.1)	83
Total	625(95.1)	4(0.6)	28(4.3)	657

ER: emergency room, removal by otolaryngologist in the emergency room; EGD: esophagogastroscopy, removal by physician using esophagogastroscopy; OR: operating room, removal under general anesthesia in the operating room.

**Table 6:** Complications.

Age	<2 Years (%)	2–5 Years (%)	>5 Years (%)	Total (%)
Complications	1 (1.0)	0 (0.0)	3 (0.5)	4 (0.3)
Total	99	672	514	1285

## Data Availability

The data presented in this study are available on request from the corresponding author.
